# Beyond comorbidities, sex and age have no effect on COVID-19 health care demand

**DOI:** 10.1038/s41598-022-11376-5

**Published:** 2022-05-05

**Authors:** Jorge M. Mendes, Helena Baptista, André Oliveira, Bruno Jardim, Miguel de Castro Neto

**Affiliations:** NOVAIMS, Universidade Nova de Lisboa, Campus de Campolide, 1070-312 Lisbon, Portugal

**Keywords:** Health policy, Health services

## Abstract

This paper explores the associations between sex, age and hospital health care pressure in the context of the COVID-19 pandemic in Portuguese mainland municipalities. To represent the impact of sex and age, we calculated COVID-19 standardised incidence ratios (SIR) in Portuguese mainland municipalities over fourteen months daily, especially focusing on the Porto metropolitan area. A daily novel indicator was devised for hospital health care pressure, consisting of an approximation to the ratio of hospitalisations per available hospital medical doctor (HPI). In addition, 14-day incidence rates were also calculated daily (DIR14), both as an approach and an alternative to the current national pandemic surveillance indicator (which is not calculated with such regularity). Daily maps were first visualised to evaluate spatial patterns. Pearson's correlation coefficients were then calculated between each proposed surveillance indicator (SIR and DIR14) and the HPI. Our results suggest that hospital pressure is not strongly associated with SIR (*r* = 0.34, *p* value = 0.08). However, DIR14 bears a stronger correlation with hospital pressure (*r* = 0.84, *p* value < 0.001). By establishing the importance of tackling sex and age through the inclusion of these factors explicitly in an epidemiological monitoring indicator, and assessing its relationship with a hospital pressure indicator, our findings have public policy implications that could improve COVID-19 incidence surveillance in Portugal and elsewhere, contributing to advancing the management of potential pandemics in the near future, with a particular focus on local and regional territorial scales.

## Introduction

By the end of 2019, a large number of pneumonia cases was reported in the Wuhan region of China, expanding rapidly to the rest of the world. This disease was named COVID-19 and its causative agent (SARS-CoV-2) was soon identified as belonging to the family of coronaviruses.

Transmission of SARS-CoV-2 between people occurs mostly through the respiratory tract (droplets and aerosols) and by contact as well.

The incubation period varies between 2 and 14 days, and patients with COVID-19 may experience a wide range of symptoms. Some of these are mild, like fever, cough, fatigue, diarrhoea, or muscle pain. Yet, much more serious symptoms appear as the disease gets worse, with people of all ages at risk of developing an intense fever and/or a cough linked with breathing difficulties or shortness of breath. Pain related to chest pressure or loss of speech or movement may also happen.

The severity of symptoms depends on a range of factors such as ethnicity, sex, pregnancy, certain medical conditions and the use of certain drugs, poverty and overcrowding, and certain professional occupations^1^.

Furthermore, patients with certain pre-existing medical conditions are at higher risk of developing severe outcomes of COVID-19, defined as hospitalization, admission to the intensive care unit (ICU), intubation or mechanical ventilation, or death. Some types of Chronic Lung disease, Heart conditions (such as heart failure, coronary artery disease, or cardiomyopathies), Cerebrovascular disease, Chronic kidney disease and Cancer are amongst these medical conditions^2^.

Several types of Epidemiological surveillance have been proposed and applied to control the COVID-19 pandemic. This study focuses on applying *comprehensive routine surveillance*, a form of surveillance that assumes complete testing of all suspected cases. For this purpose, the number of newly confirmed cases/100,000 inhabitants is used, an indicator that depicts the rate at which new events occur in a population and can also be designated as the Incidence Rate^3,4^. This form of epidemiological surveillance provides the most accurate approximation to the intensity of COVID-19. In addition, it provides the capability of measuring the geographic evolution and severity of this disease. Furthermore, it is also helpful to keep track of temporal trends and compare areas within the same country^3^, such as municipalities.

As the COVID-19 situation evolved in Portugal, from March 2020 up to the present, the preferred indicator for this type of surveillance (and to report the *status quo* to the population) stabilised mainly on the *Incidence Rate* per 100,000 inhabitants over 14 days, reported at the municipality level^5^ with a 14-day interval. However, this strategy has been criticised as being inefficient for decision-making due to the considerable time lag between incidence reports which may lead to delays in pandemic management decisions at the local level, and provide the general public with non-updated information. Daily reporting is released at the NUTS (Nomenclature of Territorial Units for Statistics) level 2, though, but these are basic regions for the application of regional policies^6^ that encompass a large number of municipalities, thus providing a generalized regional overview, with low territorial granularity. For instance, NUT2 *Centro* (Center of mainland Portugal) comprises 100 municipalities^7^.

A different insight on the evolution of COVID-19 municipal incidence may be obtained by the *Standardised Incidence Ratio* (SIR). This indicator represents the ratio of the incident number of cases of a health condition within the study population to the expected incident number if the study population experienced the same incidence rate as a standard population (or other) for which the incidence rate is known^4,8^.

Regardless of the applied indicator, it is essential to benchmark its accuracy. Towards this purpose, one of the most critical consequences of increasing incidence is the risk of proportionally increasing pressure over national health systems, more specifically over hospital response capacity. In effect, the concentration of large numbers of severely ill patients in a narrow period nearly overwhelmed hospital capacity, and the impact of the novel coronavirus on the health and safety of patients, staff, and healthcare organizations, has not yet been accurately assessed. As more significant numbers of patients infected with COVID-19 are admitted to healthcare premises, conventional practises for providing safe and quality care face challenges with patient isolation protocols, lack of family presence, and staff limitations regarding the availability of personal protective equipment^9,10^.

As per our knowledge, there are not many studies analyzing the correlation between the number of cases of COVID-19 and the utilisation of health care services, but there are many studying the correlation between death rates and health care services availability or the lack of it. As an example, in France^11^, analised data from COVID-19 hospitalizations, severity (admission to intensive care units for reanimation or endotracheal intubation) and mortality, from 19 March to 8 May 2020, corresponding to the first French lockdown (all rates were age-standardized to eliminate differences in districts age structure). These authors show that the higher case-fatality rates observed by districts are mostly related to the level of morbidity. In metropolitan France, the higher case-fatality rates were generally related to the higher level of hospitalization, then potentially connected to the overload of healthcare system. In Spain^12^, showed that the Spanish regions with more rapid and extensive spread of SARS-CoV-2 had higher infection fatality rates. These findings are compatible with the theory that slowing down the spread of COVID-19 reduces the infection fatality rate and case fatality rate via preventing hospitals from being overrun, and thus allowing better and lifesaving care.

**Figure 1:**
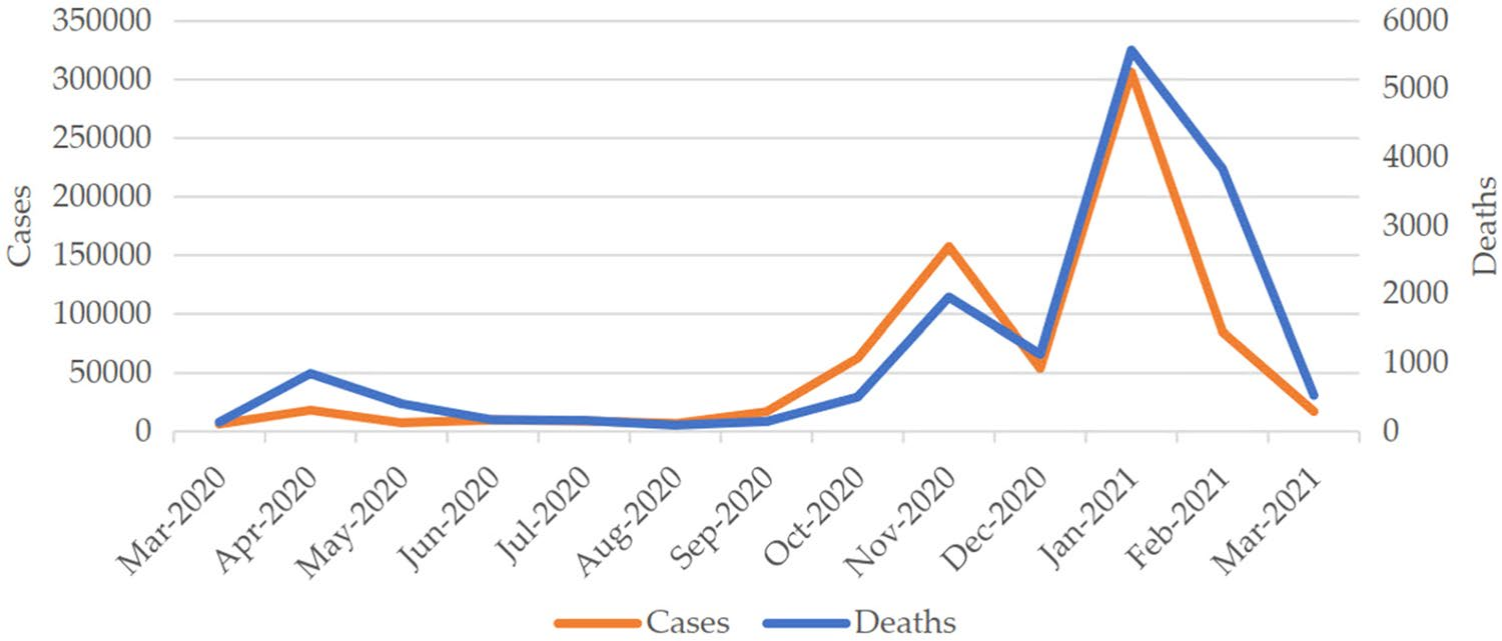
COVID-19 cases and deaths in Portugal, 1st—3rd waves. *Source*:^13^, adapted.

Ápropos the evolution of COVID-19 in Portugal throughout this study (March 2020–April 2021), the first wave started at the beginning of March 2020 and lasted just over a month. A state of emergency was in effect from 19 March 2020 to 2 May 2020. This period included several measures aimed at controlling the spread of the disease, such as the obligatory adoption of remote work, and for more than a year, progresses and setbacks were observed in some of these measures. Despite the lockdown measures it implicated, the first wave was minor in incidence compared to later ones, with the worst day registering 1516 cases on 10 April. Beyond May 2020, Portugal experienced a flattening curve and a plateau with few cases until the beginning of October. The second wave then started to unravel and reached its peak by 4 November 2020, with 7497 cases registered. The evolution of cases and deaths in Fig. 1 suggests that the third wave began without the second one having really ended.

The situation escalated after December 2020, and a new state of emergency was declared on 15 January 2021, lasting until 30 April 2021. On 28 January 2021, Portugal registered 16,432 new cases, the highest national count attained during the course of the pandemic until then. Along with the second state of emergency, a new set of control measures was deployed, and after January 2021 the incidence gradually subsided, with only 375 new cases confirmed on 25 May 2021^5,13^.

At the very beginning of this research, we asked ourselves if, besides the contribution of co-morbidities already established in the literature, the combination of patients' sex and age would contribute to hospital pressure, in other words if higher numbers of infected patients belonging to either sex, or to (putatively) higher age segments of the population would lead to increased pressure on hospital units. Hence, it was possible to hypothesize that it would.

By tackling the research issues placed above, our study aims to contribute to the improvement of comprehensive COVID-19 routine surveillance at the municipality level by testing with incidence indicators that diverge from those currently used, and benchmark the accuracy of the chosen indicators to depict incidence in the current COVID-19 surveillance context, therefore contributing to increasing the accuracy of decision-making in the context of COVID-19 crisis management.

## Materials and methods

### Research area selection

**Figure 2:**
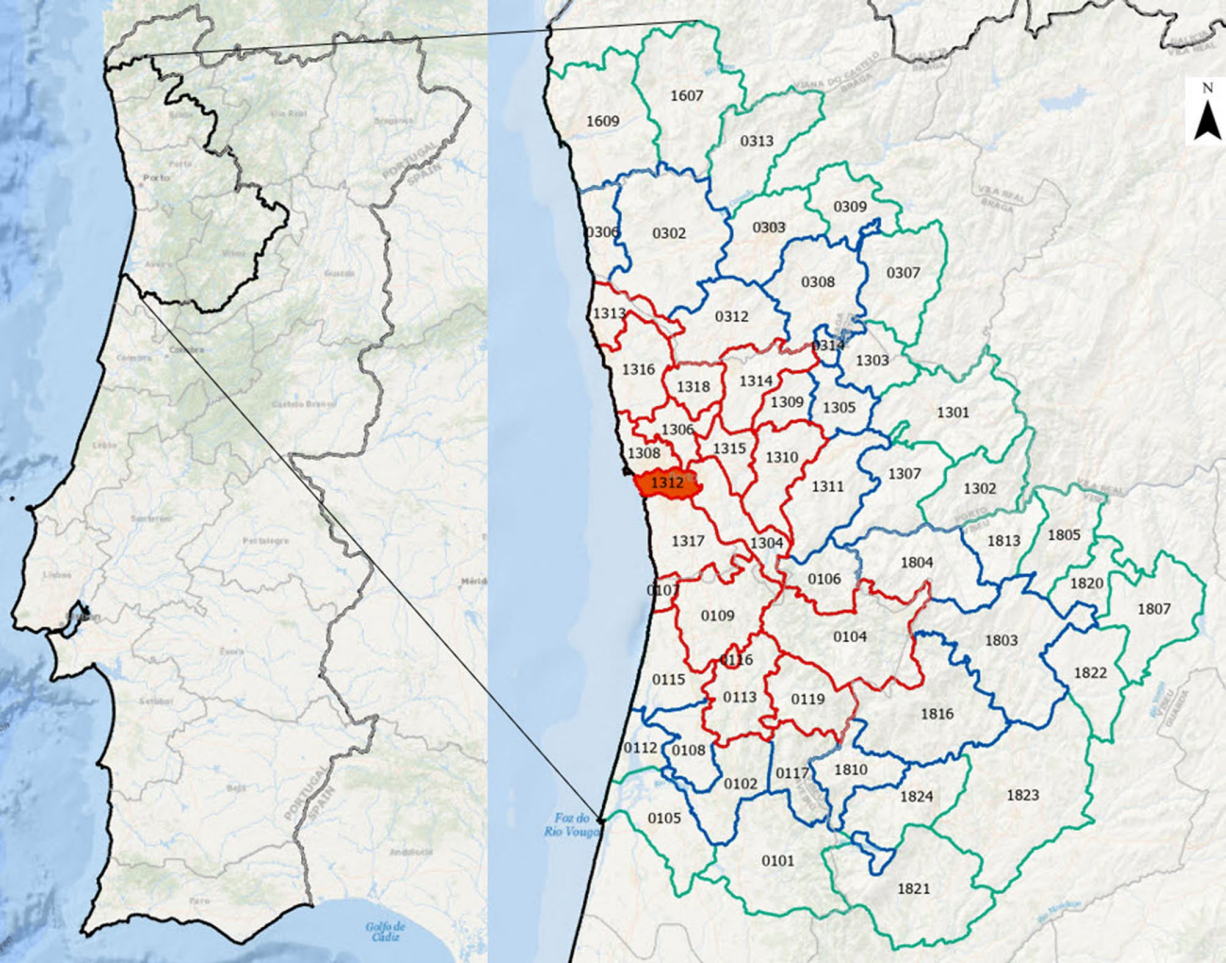
Research area. Municipalities in red: Porto Metropolitan Area; blue: adjacent neighbours (first order); green: adjacent neighbours (second order). Specific municipality data can be consulted on Annex 1, using the ID on the map.

All municipalities in the Porto metropolitan area (*Area Metropolitana do Porto—AMP*), and with its second-order adjacent neighbours, were selected as the research area (Fig. 2). The comprehensive list of chosen municipalities can be consulted in Table 1. Porto (41° 9′ 0″ N, 8° 36′ 37″ W) is the second largest metropolitan area in Portugal. AMP bears an area of 2040 km^2^, a total population of 1,722,374 inhabitants and a population density of 844 inhab./km^2^, close to that of Lisbon Metropolitan Area, with 954 inhab./km^2^^14^.

**Table 1:** List of municipalities within the research area.

ID	Name	Population density (Hab/km^2^)	Population	Area (km^2^)	Relative position to AMP
101	Águeda	137	46,075	335	Contiguous (2ª Order)
102	Albergaria-A-Velha	152	24,144	159	Contíguous (1ª Order)
104	Arouca	63	20,720	329	Área Metropolitana do Porto
105	Aveiro	399	78,734	198	Contiguous (2ª Order)
106	Castelo de Paiva	134	15,454	115	Contiguous (1ª Order)
107	Espinho	1402	29,516	21	Área Metropolitana do Porto
108	Estarreja	240	26,006	108	Contiguous (1ª Order)
109	Santa Maria da Feira	642	138,636	216	Área Metropolitana do Porto
112	Murtosa	141	10,279	73	Contiguous (2ª Order)
113	Oliveira de Azemeis	410	66,011	161	Área Metropolitana do Porto
115	Ovar	367	54,260	148	Contiguous (1ª Order)
116	São João da Madeira	2764	21,958	8	Área Metropolitana do Porto
117	Sever do Vouga	87	11,331	130	Contiguous (1ª Order)
119	Vale de Cambra	145	21,327	147	Área Metropolitana do Porto
302	Barcelos	307	116,187	379	Contiguous (1ª Order)
303	Braga	996	182,679	183	Contiguous (2ª Order)
306	Esposende	358	34,177	95	Contiguous (1ª Order)
307	Fafe	219	48,060	219	Contiguous (2ª Order)
308	Guimarães	632	152,309	241	Contiguous (1ª Order)
309	Póvoa de Lanhoso	160	21,499	135	Contiguous (2ª Order)
312	Vila Nova de Famalicão	653	131,676	202	Contiguous (1ª Order)
313	Vila Verde	205	46,911	229	Contiguous (2ª Order)
314	Vizela	968	23,897	25	Contiguous (1ª Order)
1301	Amarante	177	53,193	301	Contiguous (2ª Order)
1302	Baião	107	18,748	175	Contiguous (2ª Order)
1303	Felgueiras	487	56,422	116	Contiguous (2ª Order)
1304	Gondomar	1258	165,985	132	Área Metropolitana do Porto
1305	Lousada	487	46,755	96	Contiguous (1ª Order)
1306	Maia	1676	138,971	83	Área Metropolitana do Porto
1307	Marco de Canaveses	255	51,496	202	Contiguous (2ª Order)
1308	Matosinhos	2809	175,357	62	Área Metropolitana do Porto
1309	Paços de Ferreira	799	56,728	71	Contiguous (1ª Order)
1310	Paredes	549	86,067	157	Área Metropolitana do Porto
1311	Penafiel	329	69,772	212	Contiguous (1ª Order)
1312	Porto	5229	216,606	41	Área Metropolitana do Porto
1313	Póvoa de Varzim	764	62,784	82	Área Metropolitana do Porto
1314	Santo Tirso	498	68,055	137	Área Metropolitana do Porto
1314	Trofa	498	68,055	137	Área Metropolitana do Porto
1315	Valongo	1297	97,444	75	Área Metropolitana do Porto
1316	Vila do Conde	536	79,899	149	Área Metropolitana do Porto
1317	Vila Nova de Gaia	1784	300,472	168	Área Metropolitana do Porto
1607	Ponte de Lima	129	41,315	320	Contiguous (2ª Order)
1609	Viana do Castelo	265	84,417	319	Contiguous (2ª Order)
1803	Castro Daire	36	13,823	379	Contiguous (1ª Order)
1804	Cinfães	76	18,244	239	Contiguous (1ª Order)
1805	Lamego	150	24,895	165	Contiguous (2ª Order)
1807	Moimenta da Beira	44	9736	220	Contiguous (2ª Order)
1810	Oliveira de Frades	68	9936	145	Contiguous (1ª Order)
1813	Resende	82	10,137	123	Contiguous (2ª Order)
1816	São Pedro do Sul	44	15,403	349	Contiguous (1ª Order)
1820	Tarouca	78	7804	100	Contiguous (2ª Order)
1821	Tondela	71	26,357	371	Contiguous (2ª Order)
1822	Vila Nova de Paiva	27	4687	176	Contiguous (2ª Order)
1823	Viseu	192	97,249	507	Contiguous (2ª Order)
1824	Vouzela	50	9619	194	Contiguous (2ª Order)

In early March 2020, the AMP represented the starting point of the COVID-19 pandemic in Portugal and was also an important focus in mainland Portugal during the first three waves of this disease which are covered by the period of data used in our research. Given the importance of commuting between the centre of the metropolitan area (particularly Porto municipality) and its surrounding area, this research area was expanded to include its second-order neighbour municipalities too. Furthermore, to obtain a broader picture to compare with the research area, all Portuguese mainland municipalities were also tested within the scope of this study.

### Data acquisition

A temporal series (from 3 March 2020 to 22 April 2021) of Covid-19 daily incidence per municipality was obtained from the General Directorate of Health (*Direção-Geral de Saúde—DGS*)^15^, which releases it to authorised researchers periodically (although not daily). This data was further cleaned to include complete new cases only. Complete new cases are the ones that have the following information: date, municipality (Portuguese mainland), age and sex, totalling 740,103 cases.

Demographic data were obtained from the National Statistics institute (*Instituto Nacional de Estatística—INE*)^16^, namely 2019 estimates of total population per municipality and the corresponding age and sex structure.

The monthly count of hospitalizations per Portuguese National Health Service (*Sistema Nacional de Saúde—SNS)* hospital unit was obtained from March 2020 to February 2021, from the SNS Transparency Portal^17^, as well as the hospital location (latitude and longitude coordinates) and monthly availability (count) of health professionals, per hospital unit of the SNS. The national daily count of hospitalizations was also collected from DGS for the same study period.

Cartographic datasets with the administrative boundaries of Portuguese mainland municipalities were obtained from the General Directorate of the Territory *(Direção-Geral do território—DGT)*^18^.

### Epidemiological indicators and spatial analysis

A municipal Covid-19 accumulated 14-day incidence rate per 100,000 inhabitants (DIR14) was first calculated daily by applying a moving window to the temporal series (Eq. 1). This calculation aimed to provide the 14-day accumulated incidence rate with a much higher frequency than the indicator currently provided by DGS. For each calendar day, the incidence of new cases was calculated for the previous 14-days.

1 $${DIR(14)}_{t}= \frac{1}{{p}_{i} }*\sum_{t=0}^{t-13}{y}_{i}$$

with $i=1, \dots , 278$ municipalities, $y=$ confirmed new cases, $p=$ population and $t=0, \dots , 403$, representing respectively (1) the calendar day of 16 March 2020, the first day with 13 previous days of new cases available and (403) the last day, 22 April 2021.

Alternatively, the 14-day standardised incidence ratio (SIR) was calculated daily by applying a moving window to the temporal series (Eq. 2).

Accordingly, the following notations and/or definitions are introduced. For simplicity we omit the time index. For the aforementioned period, all calculations below are done at the *t* = day level, with the variables represented as follows:

- ${Y}_{kg}$ The number of observed cases in each *k* age and *g* sex group.
- ${p}_{kg}$ The number of people at risk in each *k* age and *g* sex group.
- ${r}_{kg}= \frac{{y}_{kg}}{{n}_{kg}}$ The observed prevalence proportion for each *k* age and *g* sex group.
- ${n}_{ikg}$ The number of people at risk in each *k* age and *g* sex group in the *i*th municipality.
- ${E}_{ikg}$ and ${y}_{ikg}$ The expected and observed number of cases for the *k* age and *g* sex group in the *i*th municipality, respectively, where ${E}_{ikg}= {r}_{kg}* {n}_{ikg}$.
- ${E}_{i}={{\sum }_{kg}r}_{kg}* {n}_{ikg}$ and ${y}_{i}^{* }= {\sum_{kg}y}_{ikg}$ The total number of expected and observed cases in the ith municipality, respectively.
  2 $$SIR_{i} = \frac{{y_{i}^{*} }}{{E_{i} }}$$

Equation (2) depicts the standardised incidence ratio, representing the risk of each *i*th municipality. A value of SIR greater (less) than one indicates that the area *i* has a higher (lower) than average disease risk. If the SIR_i_ = 1.15, it can be said that area *i* has a 15% increased risk of the disease.

In this case, $k=1, \dots , 9$, with each *k* representing a decade, so, *k* = 1 is for people up to 9 years of age and *k* = 9 is for people 80 years or older and $g=1, 2$, representing the sex group, males or females.

The accumulated 14-day incidence rate, as calculated above, was finally multiplied by 100,000 inhabitants, to facilitate direct comparison with the one currently calculated by DGS.

An index of daily pressure on SNS hospitals (Hospital Pressure Index—HPI) was developed, to benchmark the above-mentioned epidemiological indicators' relative success.

First, it was essential to establish a correspondence between hospital and municipal area. Within the context of the SNS, every hospital has an area of influence (usually specified on its website) that defines to which hospital an inhabitant is expected to be referred in case of need. This area of influence may include one or more municipalities. Therefore, the territorial boundaries of municipalities were dissolved to obtain an area of influence for each Portuguese mainland SNS Hospital, and all mainland municipalities were assigned the code of their respective area of influence. Since some municipalities of main Portuguese cities, such as Lisbon and Porto, include more than one hospital within its area, it was decided to aggregate all hospitals within the municipality area to a single influence area. Through this procedure, 37 influence areas were obtained (figure in supplementary materials), covering all mainland municipalities, and a direct match between hospital influence area and the municipality was achieved, each municipality belonging to one hospital influence area. All geoprocessing and spatial analysis operations were executed in ESRI ArcGIS Pro 2.7^19^.

The second step consisted of calculating daily HPI values. Monthly hospitalizations were aggregated by hospital influence area and then summed to obtain the total hospitalisations for each month in the Portuguese mainland. A monthly ratio was then established between the area of influence hospitalizations and the total count for the Portuguese mainland, allowing to establish the proportion of hospitalizations attributable to each area of influence. Next, this proportion of hospitalisations was multiplied by the daily national count of hospitalisations from COVID-19, consequently allowing an approximation to the daily hospitalisation count per area of influence. Finally, a ratio was established between each daily count of hospitalisations per area of influence and the available monthly count of medical doctors per area of influence (this count assumed, for the purposes of this study, to be constant along each day of the month). This final ratio is the HPI value, its rationale being that as hospitalisations rise (during the study period, primarily due to the correspondent rise in COVID-19 incidence), the number of available medical doctors to assist patients in hospitals tends to be progressively lower, and the pressure on the available medical human resources increases accordingly. HPI values were then mapped to municipalities through spatial overlay^20^, in other words by attributing an HPI value to municipalities that fall within a hospital influence area.

Accordingly, the following notations are introduced:

- $H= \sum {h}_{j}$ The number of hospitalisations per month for Portugal mainland, each month, during the considered period, with $j=1, \dots , 37$ hospital influence areas.
- ${w}_{j}=\frac{{h}_{j }}{H}$ The monthly proportion of each hospital area, compared to the national.
- Assuming the daily hospitalisations follow the same spatial distribution as the monthly and knowing the national number of hospitalisations due to COVID-19 (*H*_*t*_) in mainland Portugal, ${h}_{tj}={H}_{t}* {w}_{j}$ represents the number of hospitalisations per day and hospital area.
- Assuming medical personnel is constant across the period, *m*_*j*_ represents the number of available resources in terms of medical personnel in each of the *j* hospital areas.
- $HPI_{tj} = \frac{{h_{tj} }}{{m_{j} }}$ The daily hospital pressure index.

Regarding spatial and non-spatial data analysis, to obtain an initial comparative overview of the spatial distribution patterns of DIR14, SIR and HPI, daily choropleth maps^20^ were first produced for each of these variables for both the AMP study area and mainland Portugal, covering the entire temporal window of the study. These maps were also transformed into movie frames and a movie was then produced, allowing for a swifter visualization of the common spatiotemporal evolution of the three variables. A dashboard combining maps and line graphs for each variable was also produced using Microsoft Power BI^21^ for the Porto study area^22^ to add further data visualization analysis capabilities to our study.

After spatiotemporal visualisation, the Pearson correlation coefficient^23^ was calculated between each incidence indicator (DIR14 and SIR) and the HPI, for all mainland Portuguese municipalities, and its statistical significance was assessed. All non-spatial calculations were executed using the R statistical environment^24^.

Furthermore, the municipal population density was also calculated, based on the total municipality population in 2019 and the municipality area in square kilometres, to test the hypothesis of a potentially positive relationship between SIR and population density.

### Ethical statement

This research does not involve the use of data at the individual level, only data from public sources aggregated spatially at the level of municipality and temporally at the daily level. Furthermore, it does not involve the realization of any laboratorial experiments, focusing only on the “natural experiment”. In addition, all methods were performed in accordance with the relevant guidelines and regulations.

## Results and discussion

**Figure 3:**
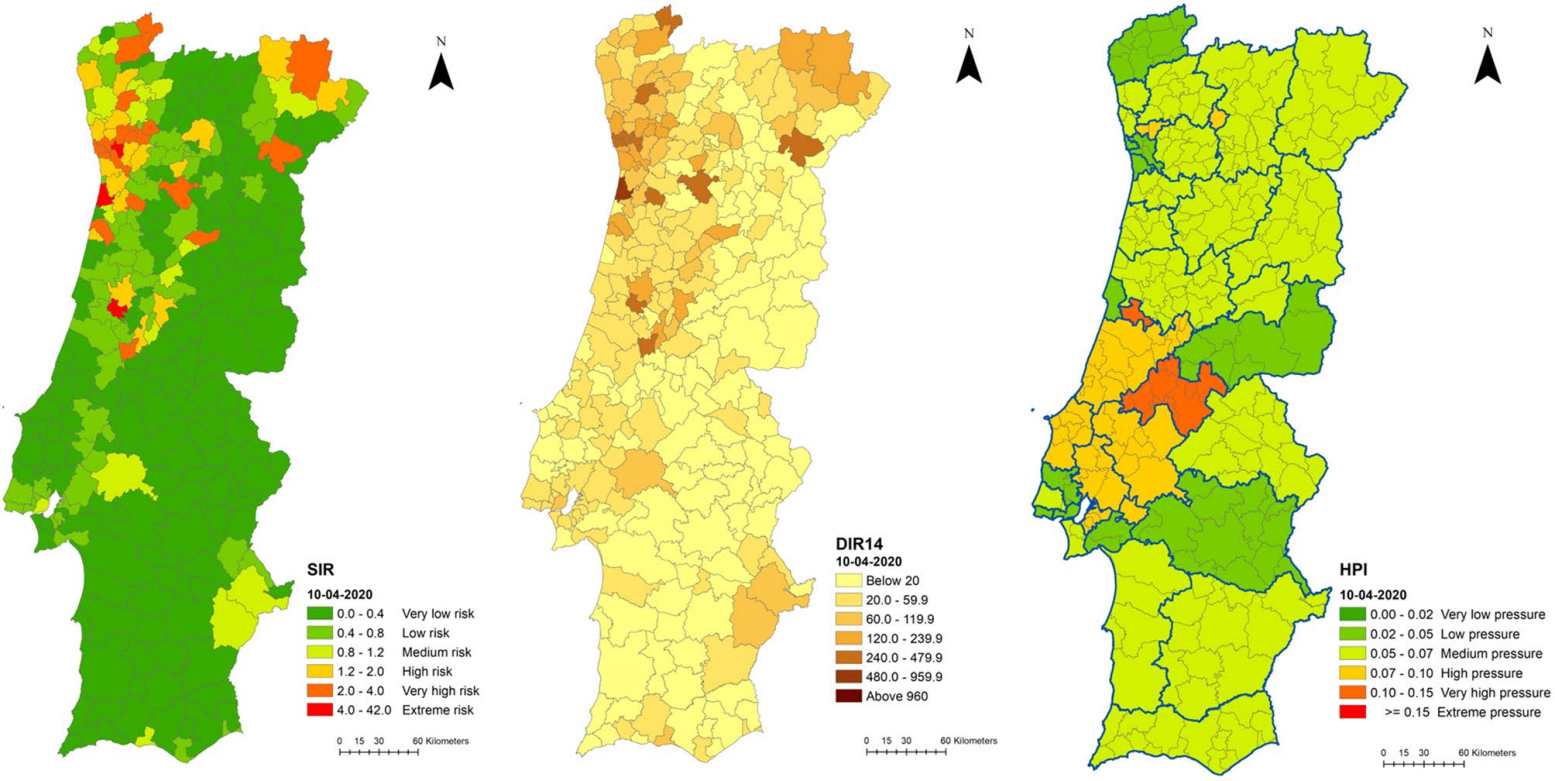
Spatial distribution of SIR, DIR14 and HPI, 10/04/2020, Portuguese mainland.

**Figure 4:**
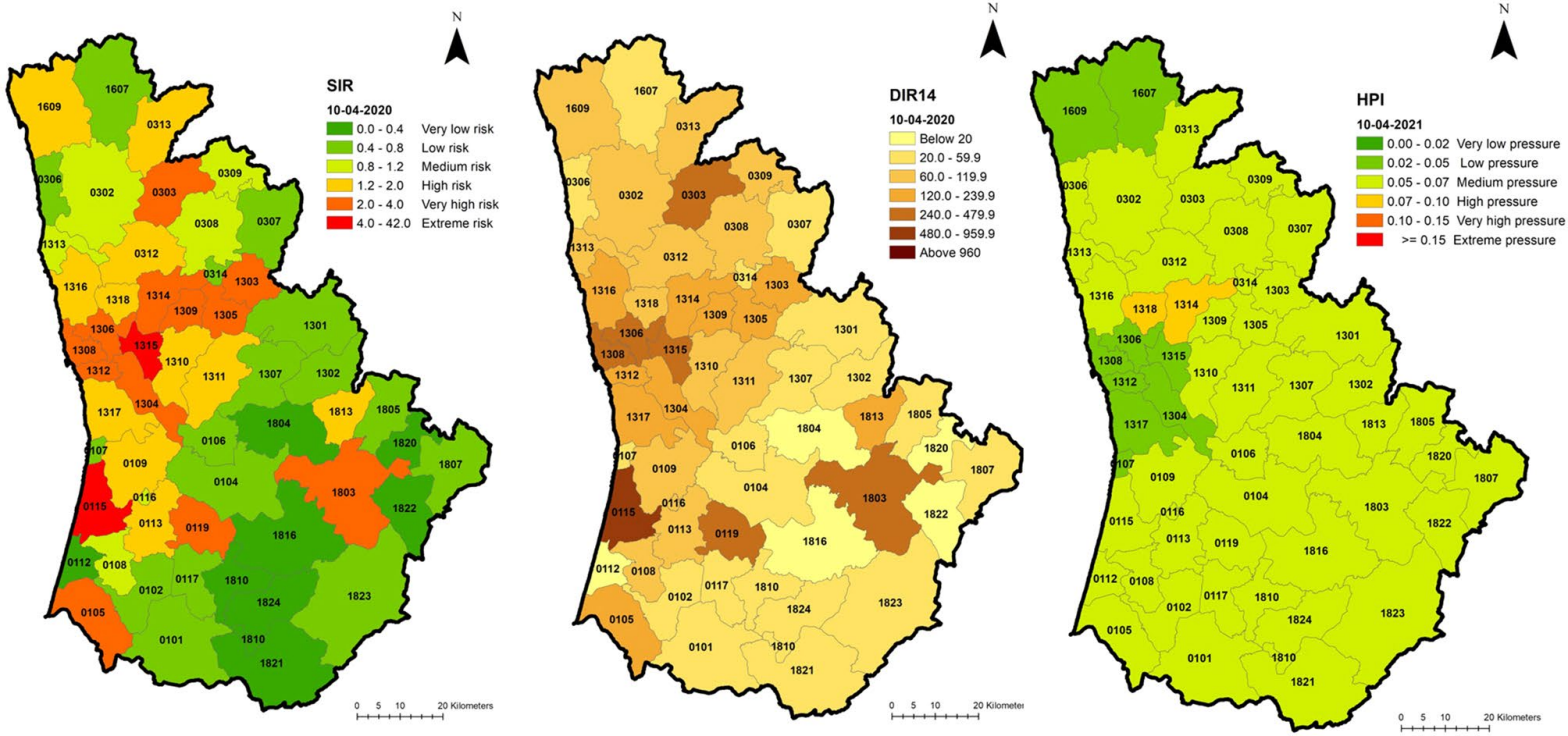
Spatial distribution of SIR, DIR14 and HPI, 10/04/2020, Porto area.

Visualising the combined spatiotemporal evolution of indicators for all Portuguese mainland suggests some alignment between indicators in time and space, but some discrepancies are also present. On the 10 April 2020 (Fig. 3), at the peak of the first wave, indicators suggested some coherence between themselves. The incidence rate (DIR14) shows a pattern of higher incidence in the northwest, with a focus on the Porto Metropolitan Area (Figs. 3, 4) but also extending to inland municipalities, and the standardised incidence rate (SIR) displays a similar pattern of excess incidence. However, the hospital pressure index (HPI) displays a different pressure pattern, with higher values occurring only in a cluster of municipalities north of Lisbon and to a much lesser extent, in the northwest near Porto. This aspect is hardly surprising, considering that the first wave exerted less pressure on the SNS, than the second and third waves.

On 9 August 2020 (figures in supplementary materials), with the disease experiencing a plateau of comparingly lower incidence between the first and second waves, the spatial patterns of DIR14 and SIR appear similar, especially in the area extending from Lisbon towards the northeast. As expected, pressure upon SNS hospitals (HPI) remained low due to the plateau of lower incidence, even considering the prevalence of active cases in need of extended hospitalization combined with the continuous incidence (albeit in relatively low numbers).

On 4 November 2020, when the peak of the second wave was attained, DIR14 and HPI displayed more similar patterns, while SIR signalled a strong cluster of higher risk municipalities around the Metropolitan Area of Porto and along the northeast border (figures in supplementary materials).

**Figure 5:**
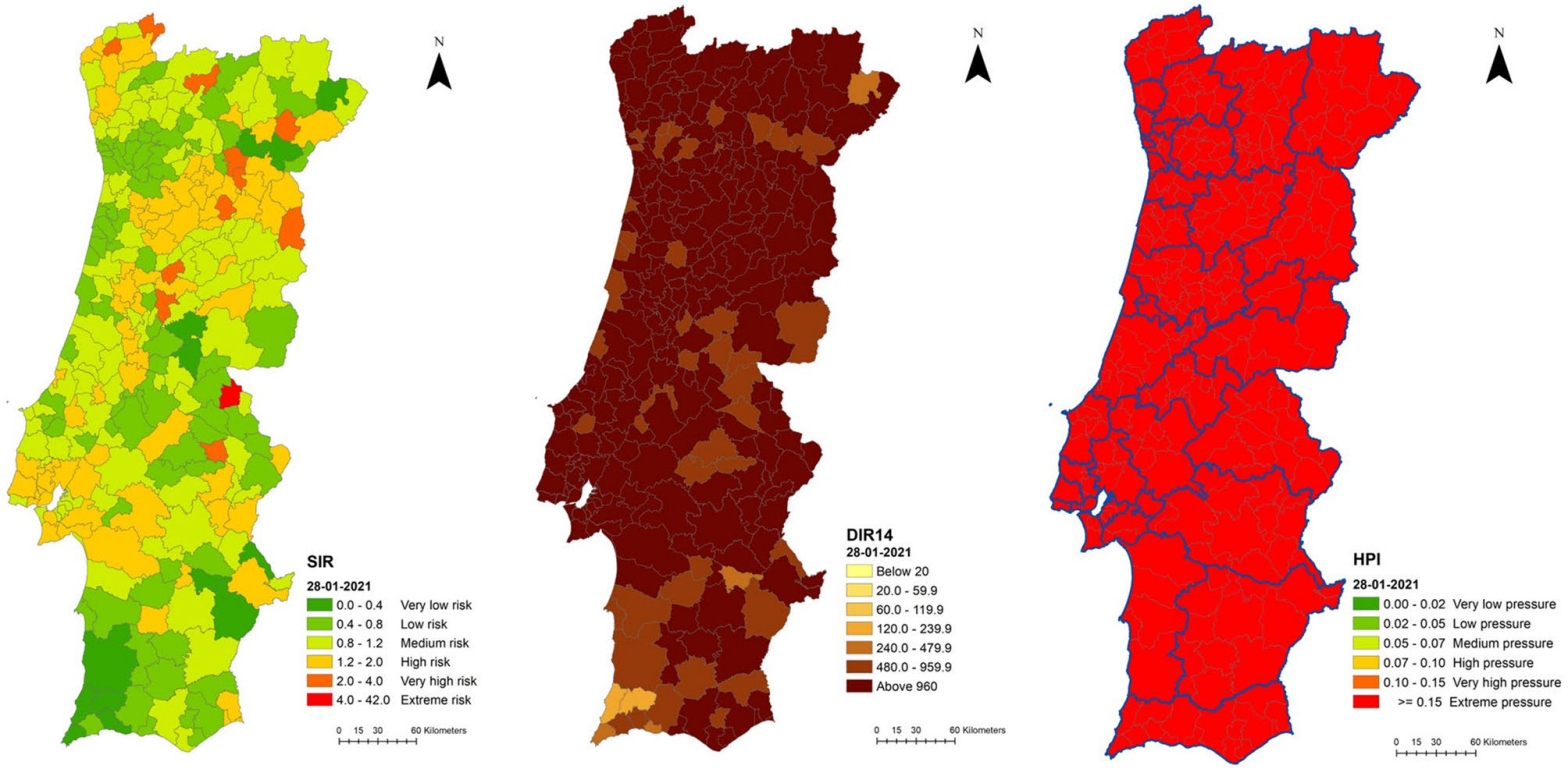
Spatial distribution of SIR, DIR14 and HPI, 28/01/2021, Portuguese mainland.

**Figure 6:**
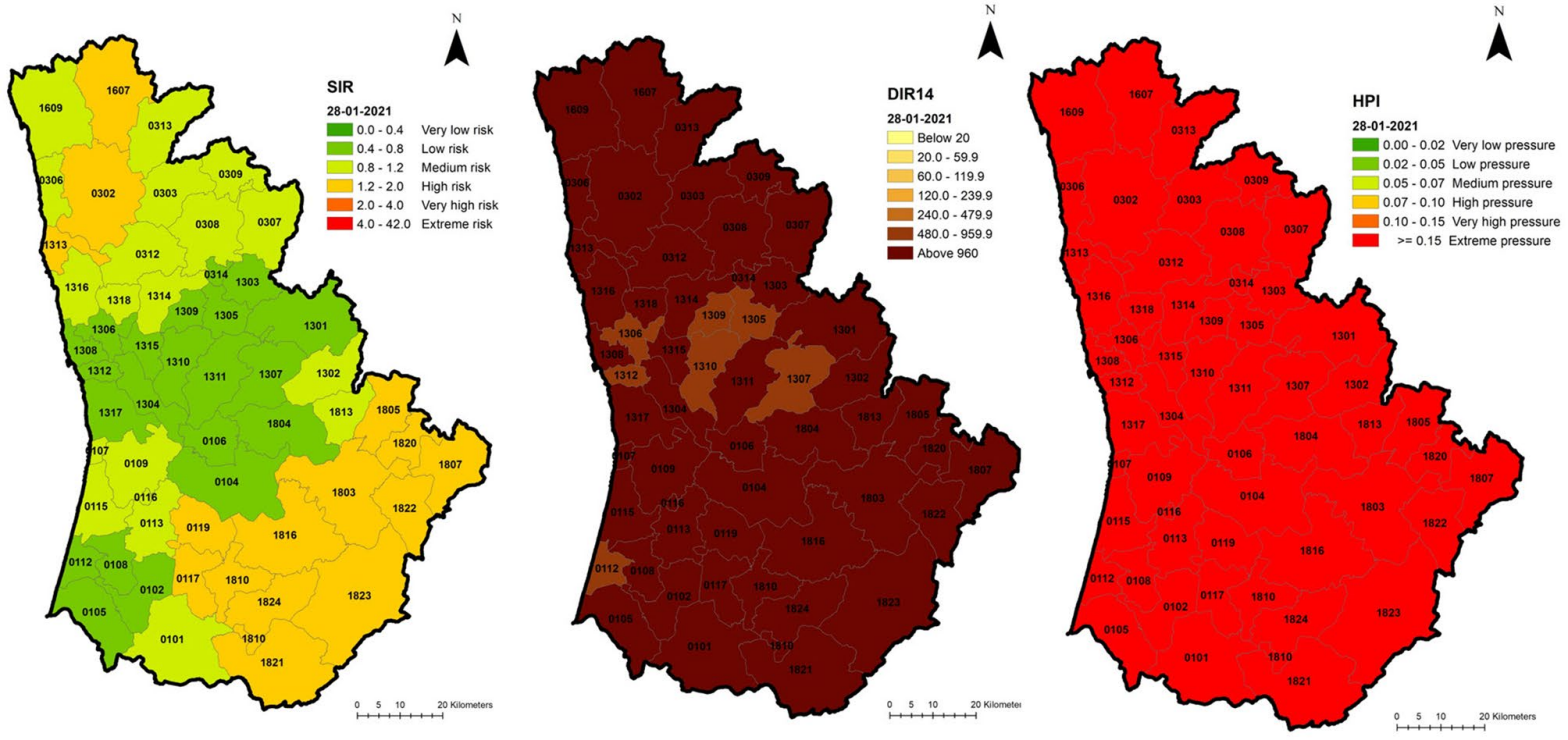
Spatial distribution of SIR, DIR14 and HPI, 28/01/2020, Porto area.

And in 28 January 2021, the height of the third wave was reached, the most critical moment in pandemic management for SNS Hospitals. As expected, with maximum pressure being exerted over the Portuguese hospital system, HPI displays all Portuguese mainland at extreme risk and DIR14 exhibits patterns of very high values in most of the territory. As SIR is a comparative measure, at this time, it can be mainly seen that the risk is across the whole of the Portuguese mainland and not concentrated in a specific area (Figs. 5, 6).

**Figure 7:**
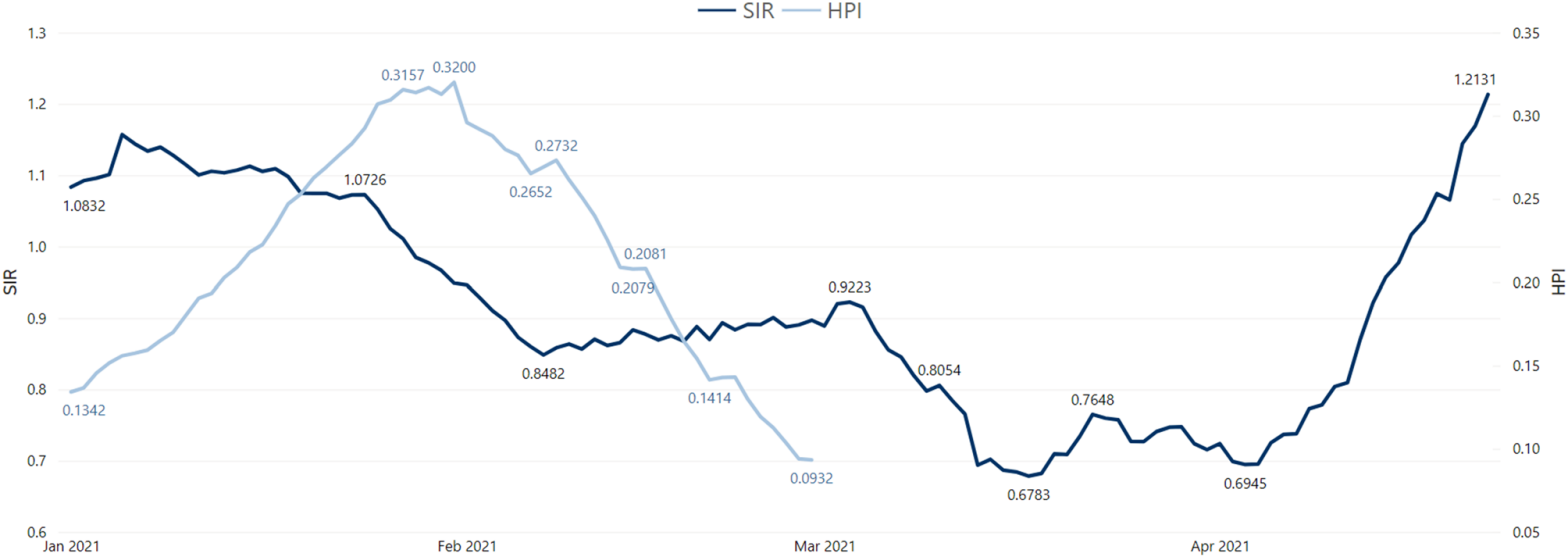
Average SIR and HPI, January-April 2021, Porto area.

**Figure 8:**
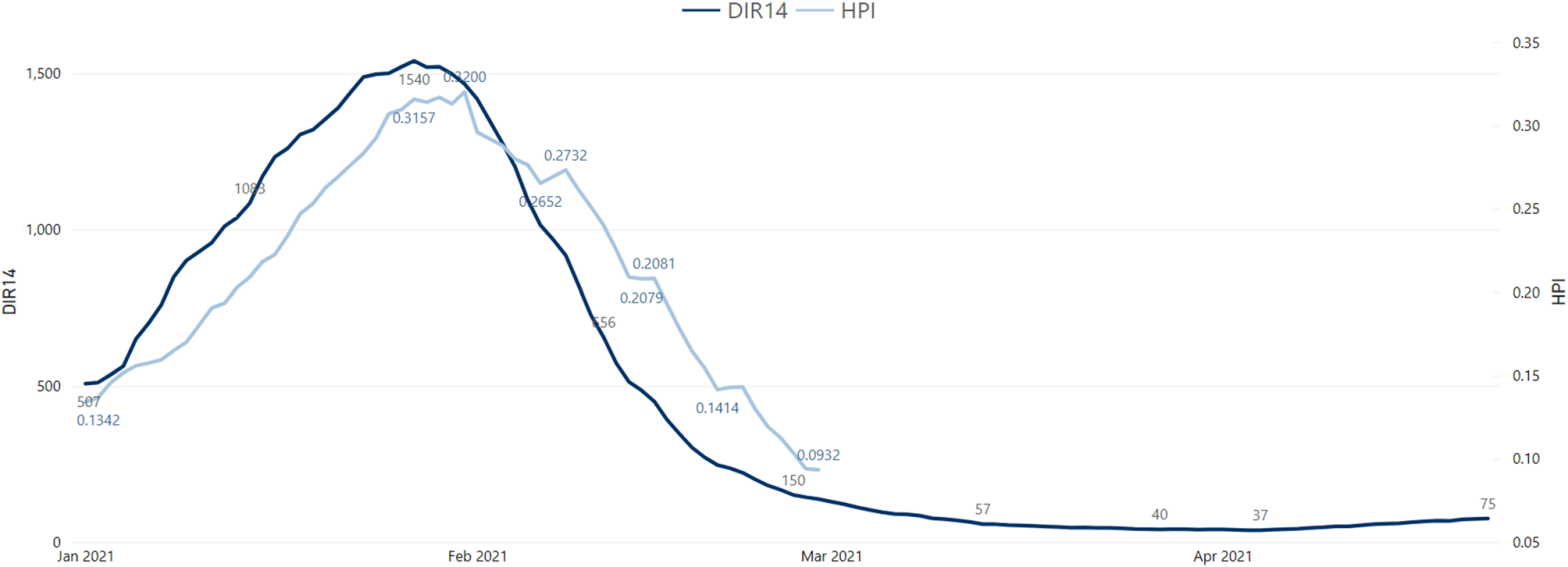
Average DIR14 and HPI, January-April 2021, Porto area.

Comparing the visual patterns is not enough to draw conclusions and therefore the correlation coefficient between SIR and HPI, on the one hand, and DIR14 and HPI, on the other hand, was calculated. Table 2 shows the minimum, maximum, mean and median correlation coefficient between these pairs, calculated from the 278 municipalities in mainland Portugal. These values represent the linear relationship between a couple of indicators contemporaneously. Alternatively, we suspected that, given the timeline between exposure to infection, case confirmation, and the need for medical care (which might vary between one to 2 or 3 weeks), there might be a lag between those two events that exists and is not being accounted for here. We also calculated correlations with a 14-day lag^25^ to test this hypothesis, whose results can be seen in Table 3. However, as correlation values became lower, this hypothesis does not seem to hold, probably due to its (arguably corrective) effect being masked by diversity across municipalities regarding their epidemiological situation. Furthermore, the curves displaying the combined evolution of the average SIR and HPI for the Porto area display lesser adherence between themselves (Fig. 7) than DIR14 and HPI (Fig. 8), as demonstrated here from January to April 2021.

**Table 2:** Minimum, maximum, mean and median Pearson contemporary correlation coefficient between SIR and HPI, and DIR14 and HPI of 278 mainland Portuguese municipalities.

	Cor (SIR_t_,HPI_t_) (*p*-value)	Cor (DIR14_t_,HPI_t_) (*p*-value)
Minimum	− 0.56 (p<0.001)	0.34 (p<0.001)
Maximum	0.91 (p=0.97)	0.97 (p<0.001)
Mean	0.34 (p=0.08)	0.84 (p<0.001)
Median	0.37 (p<0.001)	0.86 (p<0.001)

**Table 3:** Minimum, maximum, mean and median Pearson correlation coefficient with a 14-day lag between SIR and HPI, and DIR14 and HPI of 278 mainland Portuguese municipalities.

	Cor(SIR_t-13_,HPI_t_)	Cor(DIR14_t-13_,HPI_t_)
Minimum	− 0.17	− 0.23
Maximum	0.31	0.48
Mean	0.01	0.21
Median	0.01	0.21

As these correlation factors are calculated with 350 observations per municipality, a coefficient is statistically different from zero, for a 95% confidence level, if it its above(below) ± 0.1.

As mentioned above and concerning the benchmark of the tested indicators, the SIR does not seem to follow the peak moments of incidence and hospital pressure. On the other hand, the high correlation between DIR14 and HPI appears to support this conclusion. This result confirms the usefulness and good performance of DIR14 for the epidemiological surveillance of COVID-19 on a daily basis.

## Conclusions

According to^26^, based on information from WHO for more than 183 countries, Portugal's hospital resources are at par with those in Spain and Italy. The authors concluded that global COVID-19 mortality rates are likely affected by multiple factors, including hospital resources, personnel, and bed capacity.

On another publication^27^, tried to explain why Spain and Italy suffered much more than France and Germany in the COVID pandemic and attributed that to the relevant large number of patients, in those two former countries, relying on a reduced number of both hospital beds and professionals, when compared with the patients in the latter countries. The lack of hospital beds in different European countries has been attributed to a set of important cuts in the financing of public health over the last years with reduction of healthcare personnel both in the community and in the hospital care. Knowing that Portugal has experienced the same type of cuts of other European countries, early indicators of the extra need of health care services are of upmost importance. According to OECD^28^ , the Health expenditure as a share of GDP in 2019 was, respectively for Portugal, Spain, Italy, France and Germany, 9.5%/9.1%/8.7%/11.1%/11.7%. The consciousness of this reality led this research.

This study demonstrates the need to strengthen epidemiological surveillance mechanisms, particularly concerning the need for faster availability of data and information on incidence; ideally, data and information should be made available to researchers and citizens, respectively, daily and at a sufficiently disaggregated territorial scale, such as the municipality (or even below if possible, e.g. at the urban scale) and not with a delay of two weeks as is currently the procedure for DGS concerning these elements. Our results at the municipal scale stress the need for improved management of the epidemiological situation at the local and regional levels. For instance, Portugal's Northern Regional Health Administration (*Administração Regional de Saúde do Norte—ARSN*), was involved in the current study and will benefit from its results concerning municipalities within its area of intervention).

The analysis of correlation values on Table 2 unveils the misalignment between SIR and HPI spatial patterns, confirming the previous observations through visual analysis of the maps. Nevertheless, the relatively low minimum value of 0.34 for the correlation between DIR14 and HPI suggests that the course of the epidemic is far from the pressure its reference hospital experienced for some municipalities. This result reinforces the idea that a single indicator such as DIR14 should be analysed jointly with others, as long as one wants to anticipate the very short-run demand for specialised medical care.

SIR is, as mentioned, a comparative measure. Given its mathematical characteristics, it represents elevated (reduced) risk areas if its value is above (below) one. In diseases with a linear relationship between the number of cases and a certain outcome (hospitalisation, for example), an elevated (lowered) risk corresponds linearly to that outcome. COVID-19 does not have that relationship because outcomes like hospitalisations are not the same per age and sex. An elevated risk can exist if more younger people get infected, which will not be translated into more hospitalisations. Therefore, to use SIR to predict the number of hospitalisations, a “reduced” version would need to be used. A direct comparison could be made if a SIR would be calculated using only the information for the most at-risk age groups. For the time being, the average SIR for the entire period displays a pattern of elevated risk in areas with higher population density (figures in supplementary materials).

**Figure 9:**
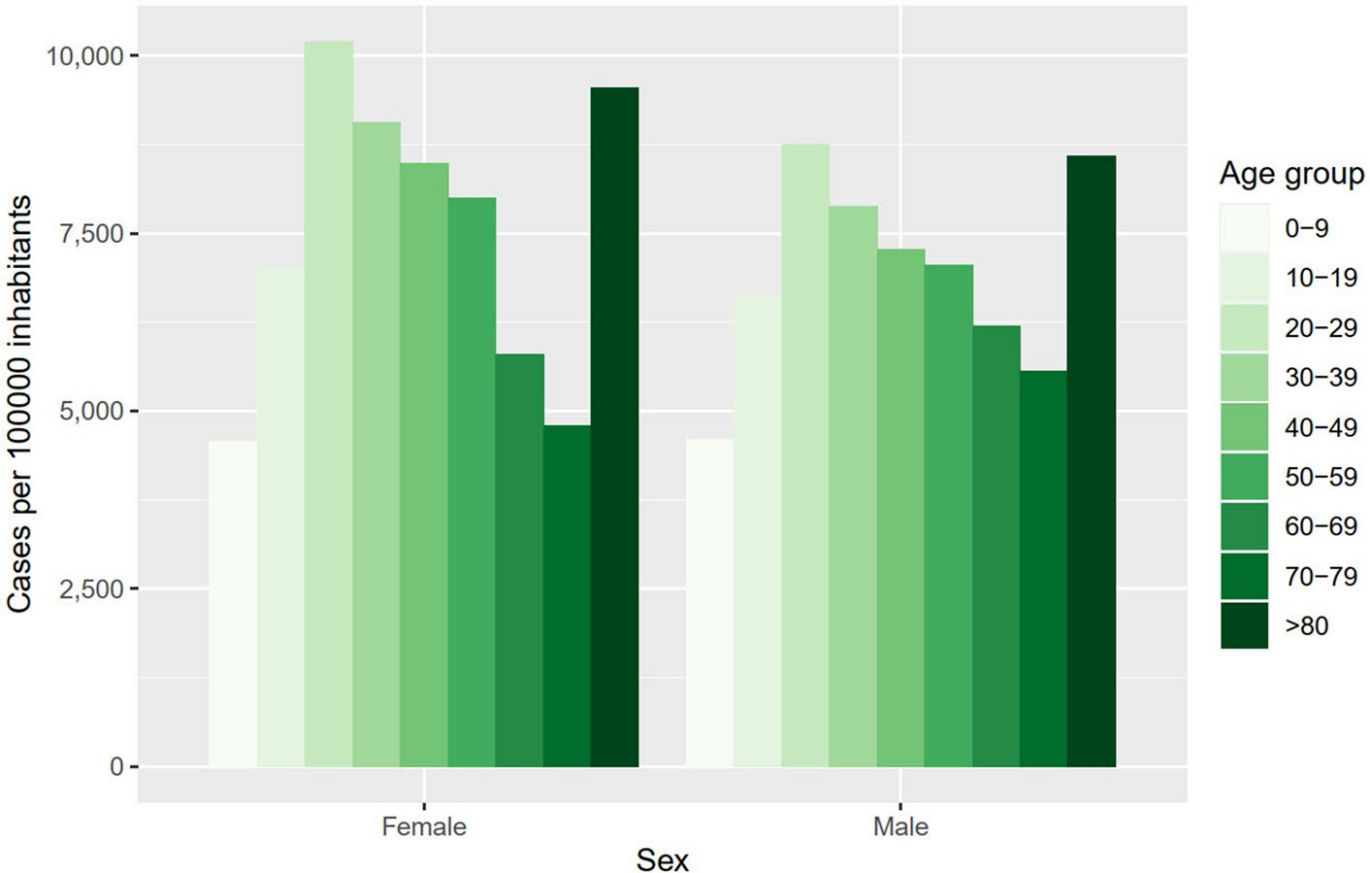
COVID-19 cases per 100,000 inhabitants during the study period, distributed per sex and age group.

Taking into account the SIR calculation methodology, which implies indirect standardisation by age structure and sex of the population, results also seem to indicate the incidence of this disease is “democratic”, in the sense that susceptibility to infection does not vary in a pronounced way with age or sex of the infected persons (although this is no longer true after infection, within the clinical course of the disease). The distribution of cases per 100,000 inhabitants between sex and age groups during the whole period of this research seems to concur with this view (Fig. 9).

However, the response of the SIR to the incidence values may still be eventually helpful to establish a comparison between the severity at municipality and country levels. It should be noted that the SIR response seemed to deteriorate, at least visually, on the days of higher incidence, and in this sense, we hypothesise that in these situations, as there is some uniformity in terms of high incidence, the response of this index tends to “dilution”. It ceases to be helpful. This possibility can be deepened in further studies, considering the hypothesis of calibrating the SIR that allows overcoming this difficulty.

The HPI could still be improved, as it was calculated using the total number of daily hospitalisations throughout the country since it was impossible to obtain a segmentation between mainland Portugal and the islands. However, we firmly believe that, even at the risk of introducing a bias towards increasing the values of approximation to daily hospitalisations by area of influence, this bias will be systematic (insofar as it is distributed equally among the areas of influence of hospitals in Portugal). It will be of negligeable magnitude, considering the size of the island area population compared to mainland Portugal's population.

These and other improvements in epidemiological surveillance processes are critical, not only for managing the current pandemic, but also for managing potential pandemics in the near future.

## Supplementary Information

Supplementary Information 1.

Supplementary Information 2.

## Data Availability

All data used sources are available as supplementary materials to this article.
