# Supplementary material for: Beyond comorbidities, sex and age have no effect on COVID-19 health care demand
Source: Sci Rep. 2022 May 5;12:7356. doi: 10.1038/s41598-022-11376-5 (PMC9069423; doi:10.1038/s41598-022-11376-5)
Supplement: Supplementary file 1 — Supplementary Information 1. [file 41598_2022_11376_MOESM1_ESM.docx]

Beyond comorbidities, sex and age have no effect on COVID-19 health care demand

Supplementary materials


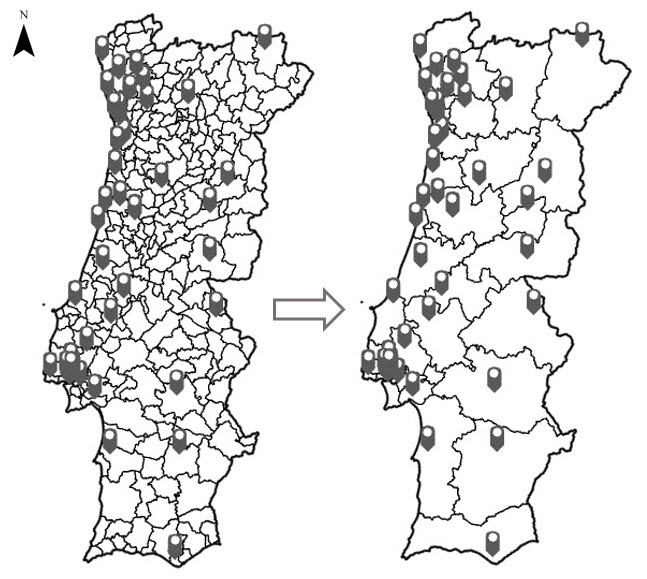


Delineation of Hospital Influence Areas by aggregation of municipalities’ areas

**
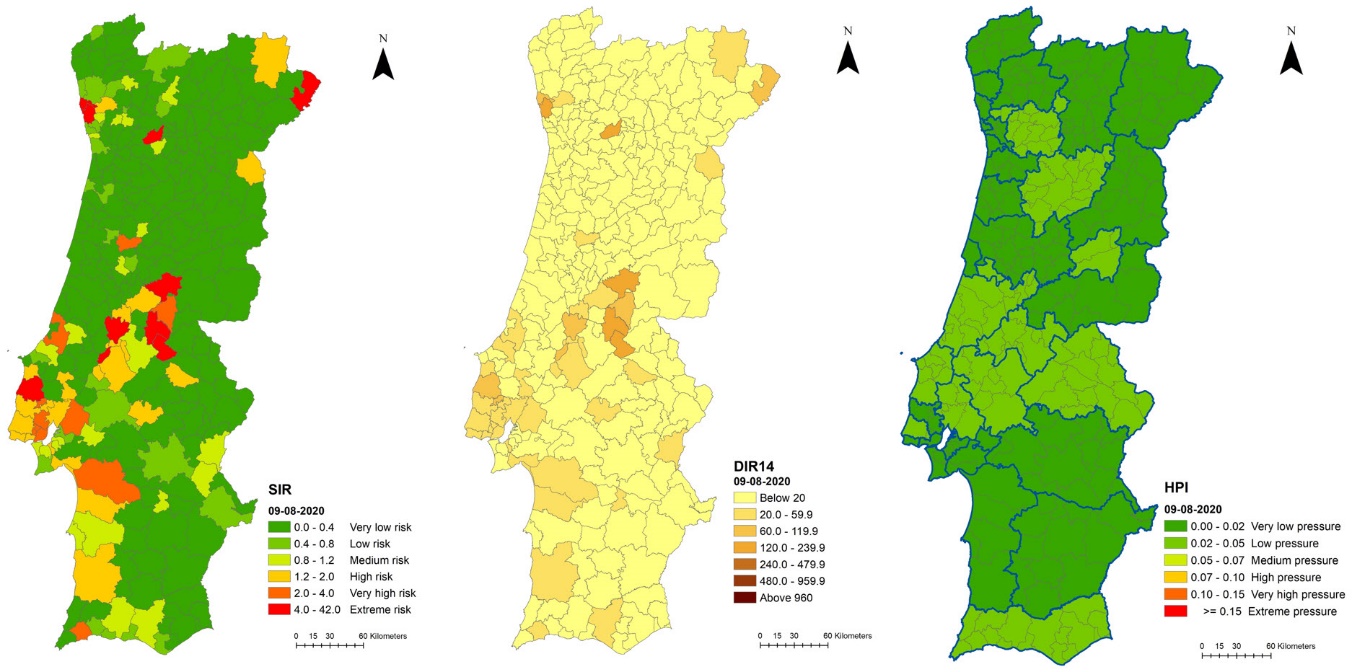
**

Spatial distribution of SIR, DIR14 and HPI, 09/08/2020, Portuguese mainland

**
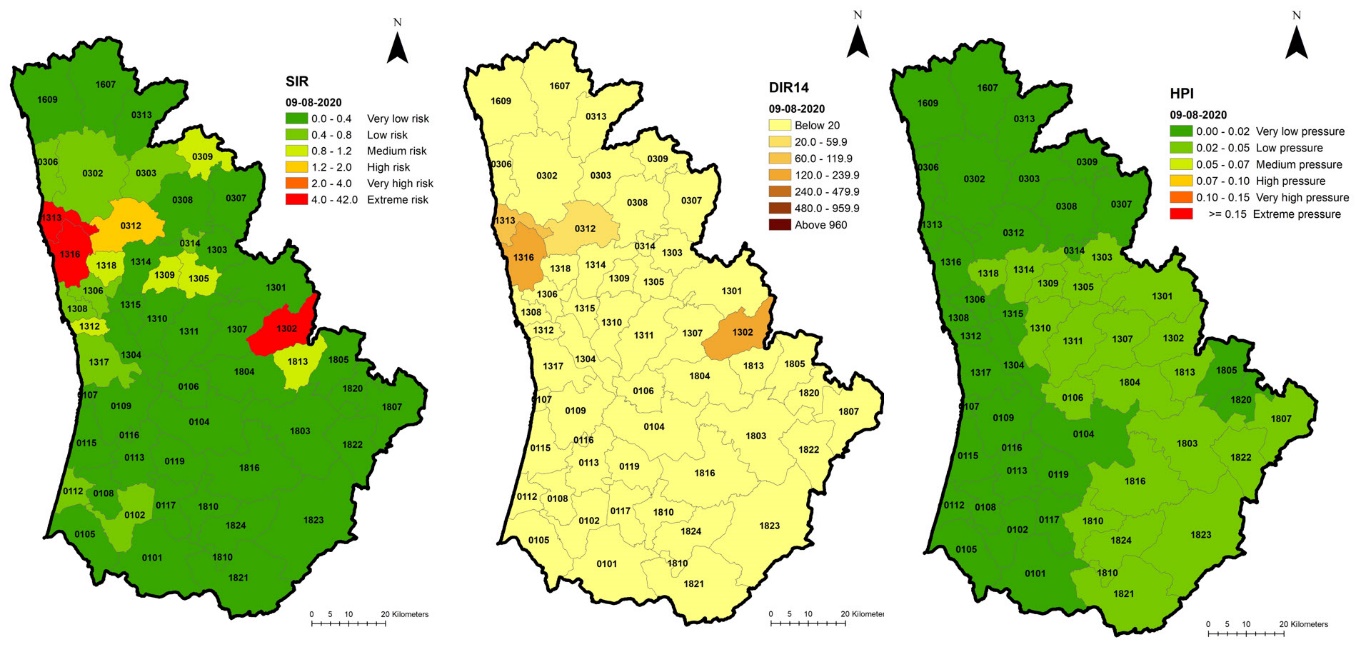
**

Spatial distribution of SIR, DIR14 and HPI, 09/08/2020, Porto area

**
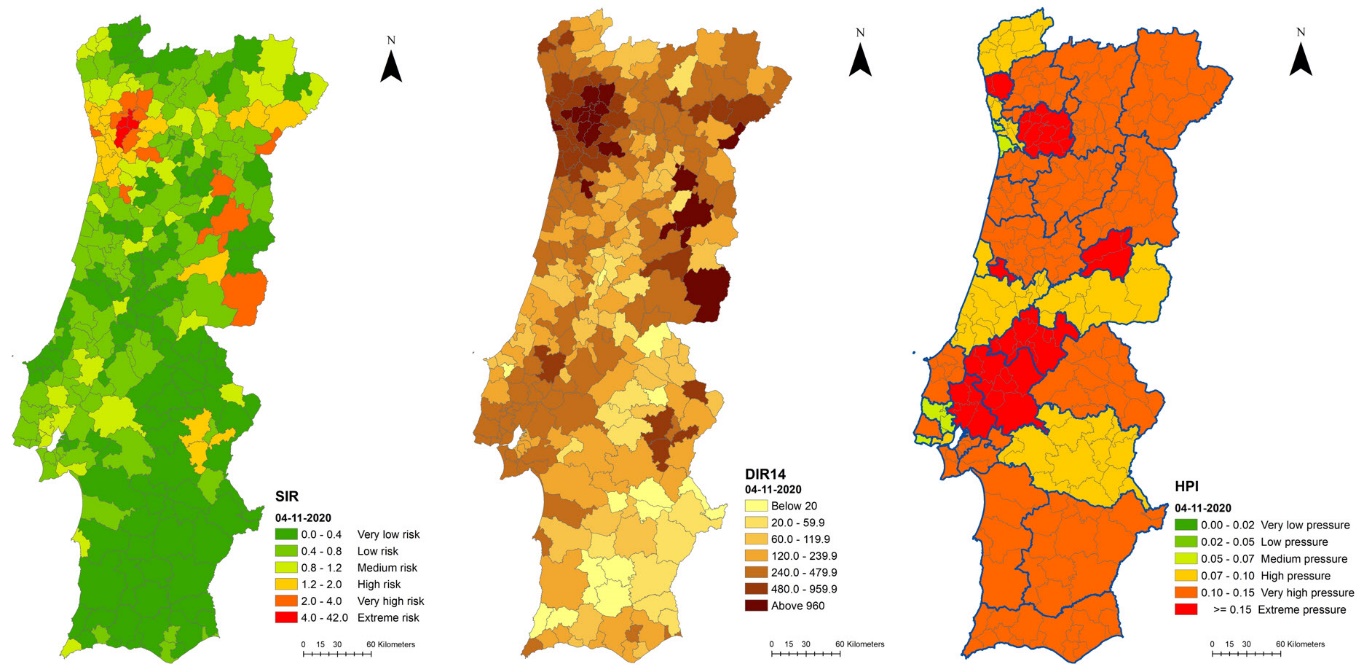
**

Spatial distribution of SIR, DIR14 and HPI, 04/11/2020, Portuguese mainland.

**
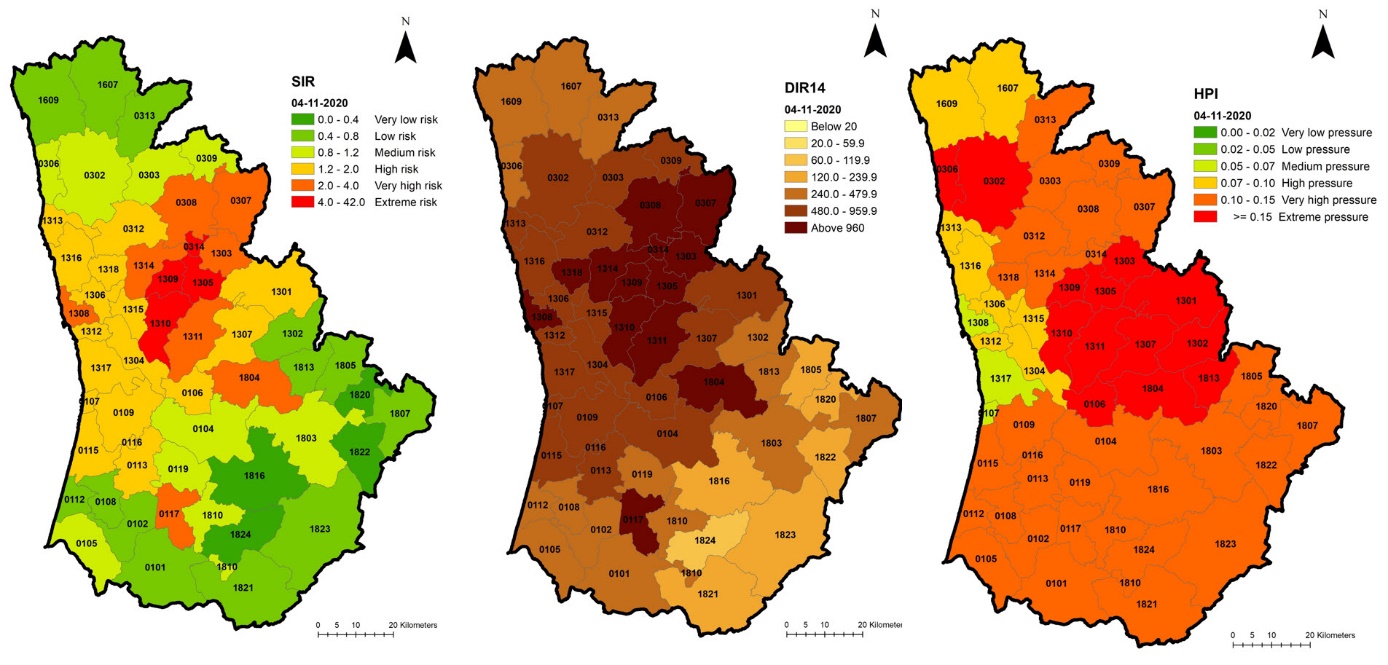
**

Spatial distribution of SIR, DIR14 and HPI, 04/11/2020, Porto area.


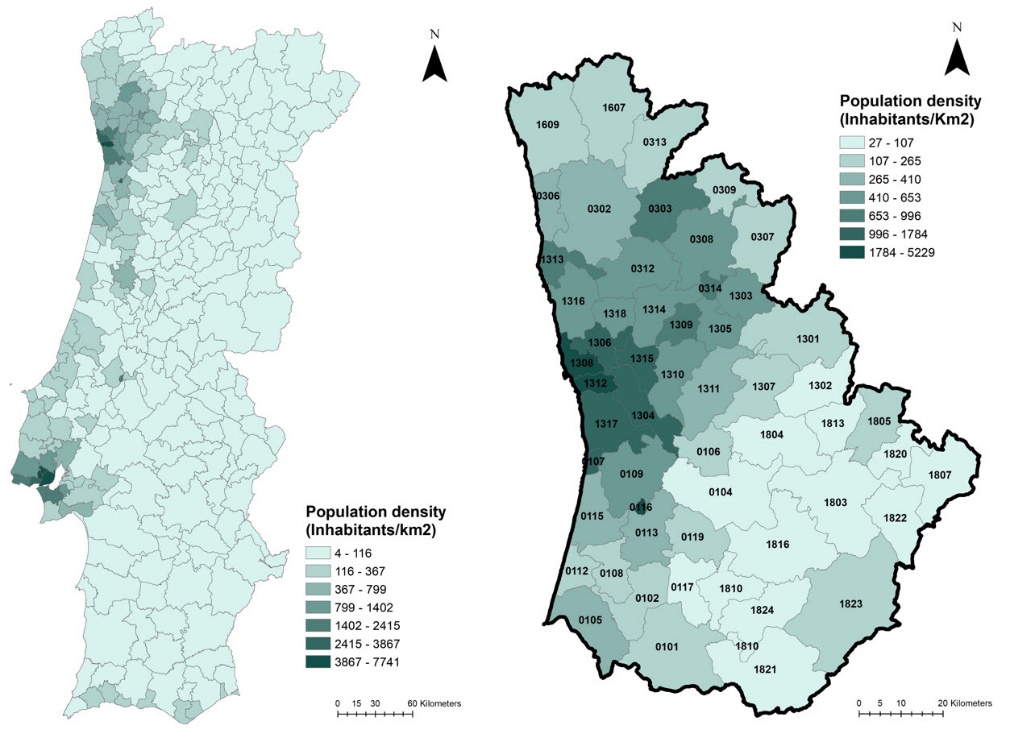


Population density (inhabitants/square km), 2019, in municipalities of the Portuguese mainland and Porto area.


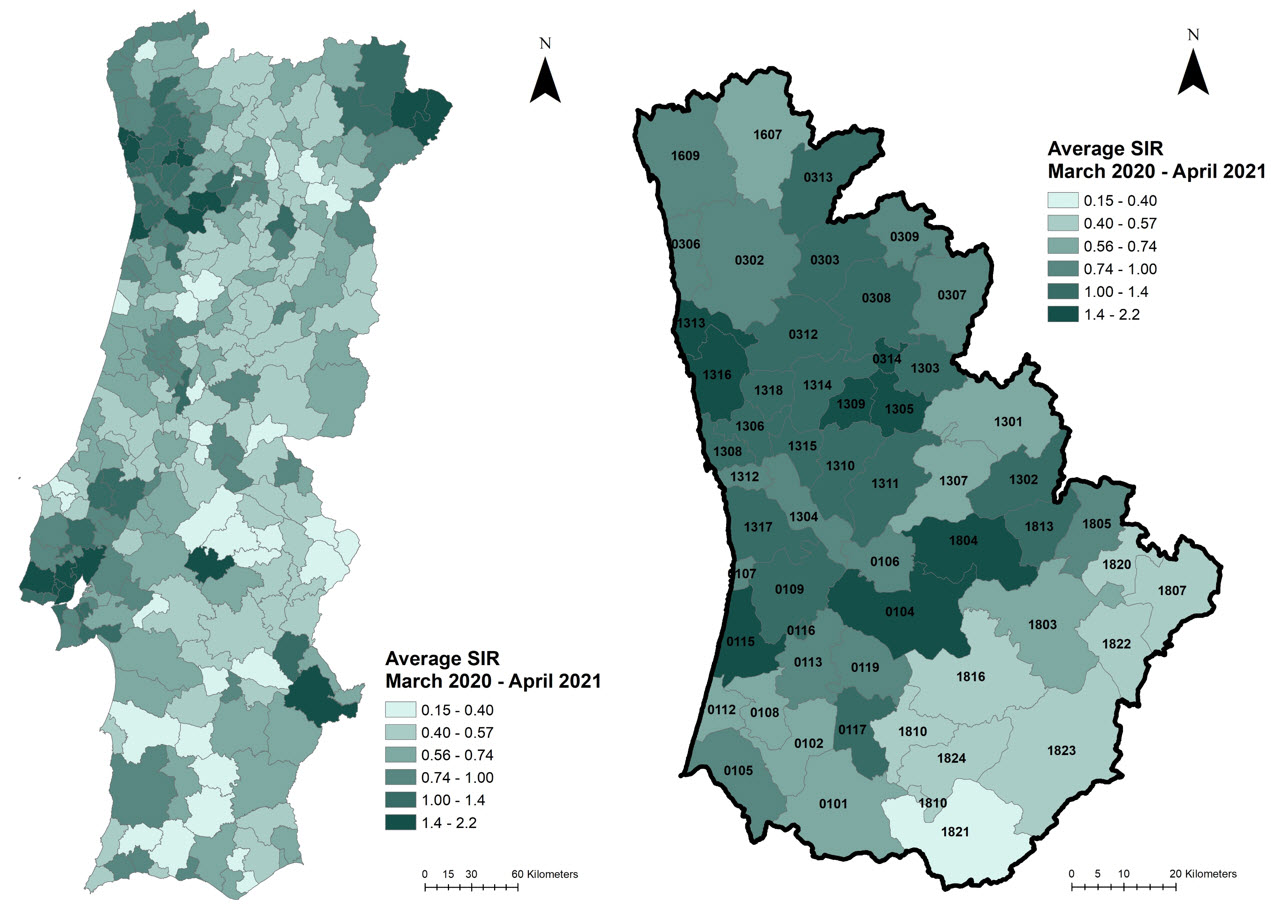


*Average SIR for the whole study period, in municipalities of the Portuguese mainland and Porto area.*
